# Resilience and immunity

**DOI:** 10.1016/j.bbi.2018.08.010

**Published:** 2018-08-10

**Authors:** Robert Dantzer, Sheldon Cohen, Scott J. Russo, Timothy G. Dinan

**Affiliations:** a The University of Texas MD Anderson Cancer Center, Houston, TX 77005, USA; b Department of Psychology, Carnegie Mellon University, Pittsburgh, PA 15213, USA; c Department of Neuroscience and Friedman Brain Institute, Icahn School of Medicine at Mount Sinai, One Gustav L. Levy Place, New York, NY 10029, USA; d APC Microbiome Ireland and Dept. of Psychiatry, University College Cork, Ireland

**Keywords:** Resilience, Stress, Depression, Immunity, Inflammation, Personal control, Optimism, Positive affect, Social support, Gut microbiota, Gut-brain axis, Psychobiotics, Recovery, Cytokines, CD8 T cells, Indoleamine 2,3 dixoygenase

## Abstract

Resilience is the process that allows individuals to adapt to adverse conditions and recover from them. This process is favored by individual qualities that have been amply studied in the field of stress such as personal control, positive affect, optimism, and social support. Biopsychosocial studies on the individual qualities that promote resilience show that these factors help protect against the deleterious influences of stressors on physiology in general and immunity in particular. The reverse is also true as there is evidence that immune processes influence resilience. Most of the data supporting this relationship comes from animal studies on individual differences in the ability to resist situations of chronic stress. These data build on the knowledge that has accumulated on the influence of immune factors on brain and behavior in both animal and human studies. In general, resilient individuals have a different immunophenotype from that of stress susceptible individuals. It is possible to render susceptible individuals resilient and vice versa by changing their inflammatory phenotype. The adaptive immune phenotype also influences the ability to recover from inflammation-induced symptoms. The modulation of these bidirectional relationships between resilience and immunity by the gut microbiota opens the possibility to influence them by probiotics and prebiotics. However, more focused studies on the reciprocal relationship between resilience and immunity will be necessary before this can be put into practice.

## 1. Introduction

Resilience is the process that allows individuals to adapt to adverse conditions and recover from them. This process is favored by individual qualities that have been amply studied in the field of stress such as personal control, positive affect, optimism, and social support. The vast literature on the relationship between stress and immunity (Cohen et al., 2001; Dantzer, 1997; Dantzer and Kelley, 1989; Dhabhar, 1998; Herbert and Cohen, 1993; Irwin, 1994; Marsland et al., 2002; Segerstrom, 2010; Stefanski, 2001; Dantzer, 2018) shows that stress can impact various aspects of immune function and, in this way, potentially modulate resistance to disease. Types of stressors that are particularly potent include enduring interpersonal conflicts and losses, economic problems, and early life adversity and trauma (Cohen et al., 1998). Although accumulating data suggest that a range of psychological factors help protect people from stress-elicited self-reported distress, less is known about the potential for resilience to buffer stress-elicited changes in physiology including immune responses. Often neglected in the discussion of resilience and immunity is that the relationship between stress and immunity is not unidirectional as immune mediators can influence factors that contribute to resilience and stress outcomes (Dantzer, 2018). Both human and animal studies show that immune mediators influence the way the brain processes information and responds to it both physiologically and behaviorally (Dantzer et al., 2008). This includes the potential influences of the immune system on emotional and behavioral factors that can contribute to resilience. The primary objective of the present review paper is to critically examine the available knowledge concerning the bidirectional relationship between resilience and immunity. A secondary goal is to discuss evidence on the potential for resilience being modulated by nutritional factors including pro- and pre-biotics. The information presented in this review builds on an expert discussion workshop that was organized by Danone Nutricia Research and took place in New York on April 1, 2017. However, the opinions expressed in this review are only those of the authors.

## 2. Definition of resilience and its measurement

In order to examine the relationship between resilience and immunity, it is important to understand what resilience exactly refers to in human and animal studies, how it measured, and how it relates to personality factors and coping styles.

The American Psychological Association (APA) defines resilience as “the process of adapting well to adversity, trauma, tragedy, threats or significant sources of stress – such as family and relationship problems, serious health problems or workplace and financial stressor” (Association). By adding the notion that resilience is ordinary, not extraordinary, APA positions resilience as part of the normal trajectory of an individual confronted to adversity while at the same time recognizing that this process can be associated with considerable emotional distress. “In either case, resilience is a not a trait that people have or do not have. It involves behaviors, thoughts and actions that can be learned and developed by anyone” (Association).

In the scientific literature, resilience is often given a more restricted meaning. In the context of stress, the term resilience usually refers to “an individual’s ability to limit or preclude the detrimental effects of a stressor” (Anisman, 2015). In medicine, psychological resilience is often distinguished from physical resilience. Physical resilience refers to the ability to recover or optimize function in the face of a disease or an acquired disability. This concept is commonly used in the aging literature (Whitson et al., 2016). In this article we will limit ourselves to psychological resilience in the sense it is defined by the APA (Association).

Definitions of resilience become important when it comes to measure it. For instance, the Resilience and Healthy Ageing Network funded by the UK Cross-Council Program for Life Long Health and Wellbeing defined resilience as “the process of negotiating, managing and adapting to significant sources of stress or trauma. Assets and resources within the individuals, their life and environment facilitate this capacity for adaptation and bouncing back in the face of adversity. Across the life course, the experience or resilience will vary” (Network). This definition of resilience is very much in agreement with the concept of a road to resilience as proposed by the APA. However, it implies at the same time that measuring the different facets of the resilience process is most likely a difficult enterprise. This certainly explains why several psychologists are taking a short cut and try to define the psychological characteristics that contribute to resilience, rather than attempting to directly measure the resilience process. As an example, Connor and Davidson (2003) state that “resilience embodies the personal qualities that enable one to thrive in the face of adversity”. The study of resilience then becomes the study of these personal qualities that can be measured in various populations independently of any specific adverse situation. Some examples of these qualities include personal control, optimism, personal competence, and spirituality. In most cases, distinct qualities are assessed individually, and tested as independent resilience factors. However, in some case (Connor and Davidson, 2003) an aggregated measure will include several qualities that are summed into a single resilience scale with those with higher scores expected to do better in the face of adversity and this independently of the source of adversity.

Human studies on resilience have been mainly correlational. They are typically conducted in natural settings and only rarely make use of well-defined laboratory stressors. They assess the natural occurrence of adverse events and the resilience factors of interest. Behavioral and biological endpoints are usually measured concurrently and hence do not allow for inferences regarding the direction of causation.

In most animal studies, well-defined stressors or adverse conditions are imposed upon animals and outcomes are assessed longitudinally by measuring behavioral and biological endpoints. This allows resilience to be operationally measured by its outcome, i.e., the ability to adapt to a stressful event (Anisman, 2015). Studies can focus on the effect of behavioral factors on the biological aspects of the resilience process or, conversely, on the influence of biological factors including immunity on the resilience process. Whatever the case, resilience is conceptualized as an active process that results in a positive biobehavioral outcome, e.g., an attenuated effect of the stress procedure. Of note, this approach could be extended to human studies of resilience as well even if there are obvious limitations in the way environmental conditions and immunity can be manipulated.

For the purpose of the present review, we will consider resilience as a dynamic process whose ultimate goal is to enable individuals to achieve a favorable outcome in face of adversity thanks to a number of psychological and biological qualities that depend in part on intricate relationships between the immune system and the brain. Despite the considerable differences in the way resilience has been and continues to be studied in the human and animal literature (Table 1), we believe there is sufficient evidence to build a case for the importance of these relationships in our understanding of what makes the resilience process successful.

We will begin by examining in detail the influence of resilience factors on the effects of stress on immunity. We place special emphasis on identifying environmental and individual factors that facilitate resilience and on specifying the behavioral and biological mechanisms that link these factors to immune function. However, our objective is to go beyond this unidirectional description of the relationship between resilience and immunity and examine how basic immune processes such as inflammation can in turn result in profound adjustments in physiology and behavior that have important modulating influences on the quality and intensity of the essential psychological components of the resilience process. Additionally, we will consider how stress-induced variations in the gut microbiota can modulate the outcome of the resilience process particularly in terms of mental health. We will conclude by discussing how a better integration of human and animal studies on resilience and immunity can be achieved.

## 3. Stress, resilience and immunity: association studies

### 3.1. Psychological stress and immunity in humans

In the context of psychoneuroimmunology, resilience is only an issue to the extent that stress actually reduces the ability of the immune system to respond to challenge. As mentioned earlier, there is a large literature, including both laboratory experiments and field research addressing the roles of various stressful situations, and the perceptions of stress on the immune status of human participants. Primarily these studies have used *in vitro* assessments of specific aspects of immunity such as natural killer cell activity, and mitogen stimulated lymphocyte proliferation, or *in vivo* assessments including antibody production to vaccinations and circulating biomarkers of inflammation such as interleukin-6 (IL-6) and C-reactive protein (CRP). These literatures are reviewed elsewhere and generally suggest that *chronic* stressors are associated with suppression of both cellular and humoral measures of immunity, see reviews by Marsland et al. (2002), Herbert and Cohen, (1993) and Segerstrom and Miller (2004). This is not necessarily true for acute stressors. In general, short-term stress enhances immunoprotective processes involved in wound healing, vaccination, and response to anti-infectious and anti-cancer agents, whereas chronic stress tends to suppress these immune responses and exacerbate pathological immune responses (Segerstrom and Miller, 2004; Dhabhar, 2014). A possible mechanism for these differences will be discussed below. Importantly for the purpose of the present review, both acute and chronic stress are associated with increases in circulating inflammatory markers in human studies (see meta-analytic reviews on acute stress by Marsland et al. (2017) and Steptoe et al. (2007), and earlier conclusions on acute and chronic stress by Segerstrom and Miller (2004)).

Other work has taken a broader approach to assessing the function of the immune system. Instead of focusing on the association of stress and biomarkers of immunity, it directly assesses the association of stress and the immune system’s ability to prevent infectious disease, for an overview, see Cohen et al. (2016). In these studies, psychological stress was assessed in healthy adults who were subsequently (and experimentally) exposed to a virus that causes the common cold. They were then quarantined and followed for 5–6 days to determine who became infected (shed virus) and who developed a clinical illness as manifest by infection and objective signs (mucus production and congestion) of the cold. Generally, approximately 1/3rd of those exposed to a virus in these studies develop a clinical illness.

Using this paradigm, exposure to recent and chronic stressful life events has repeatedly been shown to increase an individual’s risk of developing clinical illness following inoculation with the challenge virus. The association increases with increased duration of the stressful event, and is most apparent for those experiencing interpersonal or financial events (Cohen et al., 1998). In these studies, the potential of multiple alternative explanations including participant age, education, sex, weight, height, or pre-existing immunity (virus-specific antibody level) were eliminated through statistical adjustment. The investigators also tested for the possibility that the association was attributable to stress-elicited elevation in levels of epinephrine, norepinephrine, and cortisol or poorer natural killer cell activity, or poor health practices like smoking, excessive alcohol consumption, poor diets, low levels of physical activity, and poor sleep. Contrary to expectations, none of these (alone or together) explained why stress was associated with greater risk of developing a cold (Cohen et al., 1998; Cohen et al., 1991).

New insights about the role of the immune system in the pathogenesis of the common cold led to a different hypothesis about how psychological stress might influence disease susceptibility. Pro-inflammatory cytokines are released by the innate immune system in response to infections. These chemical messengers elicit an inflammatory response, drawing immune cells to the infected area to help orchestrate the immune defense against the infectious agent. However, if the immune system produces *too much* of these inflammatory chemicals, the results can be toxic (Cohen et al., 1999). In the case of infection with a common cold virus, producing too much pro-inflammatory cytokine triggers cold symptoms, such as nasal congestion and runny nose.

These results raised a dilemma for the researchers. Acute stress exposures in the laboratory and natural settings had been found to *increase* circulating levels of cortisol, a glucocorticoid hormone which *normally reduces inflammation* by turning-down the release of pro-inflammatory cytokines. Yet even though acute stress was associated with increased cortisol (and hence would presumably decrease cytokine release), they found that people who suffered from chronic stress produced more, not less, pro-inflammatory cytokine (Cohen et al., 1999). In response to this apparent contradiction, they hypothesized that when people are exposed to major stressful events over a prolonged period, their bodies adapt to the initial increase in cortisol by reducing immune cell responsiveness to cortisol (a process called glucocorticoid resistance) (Cohen et al., 2012; Miller et al., 2002). As these cells become less responsive, the body loses the ability to turn-down the inflammatory response. This hypothesis was supported by a series of viral-challenge studies showing that chronic stress was associated with increased glucocorticoid resistance; that greater glucocorticoid resistance predicted an increase in the infection-triggered production of pro-inflammatory cytokines; and that greater glucocorticoid resistance predicted a higher risk of developing a cold when exposed to a common cold virus (Cohen et al., 2012).

In sum, chronic psychological stressors predicted an increased risk of developing a common cold for those exposed to a cold virus. The association between stress and disease occurs because chronic stress interferes with the body’s ability to turn-off the immune system’s production of inflammatory chemicals; and this failure in regulation (maintaining a proper level) of inflammatory response occurs because chronic stress results in immune cells becoming insensitive to cortisol.

### 3.2. Resilience factors providing protection from stress effects on immunity

Why are some individuals resilient to the pathogenic effects of stressful events? This question has been addressed by a number of animal studies that have focused on individual differences in resilience to physical or social stressors. This literature will be examined in detail in Section 4.4 as it has been at the origin of the discovery of the importance of immune factors in the resilience process. Accumulating data from human studies similarly suggest that a range of psychological factors may play protective roles against the deleterious effects of stressful events. Here we discuss four factors that have received sufficient attention to address their potential effectiveness for protecting against the physiological effects (with an emphasis on immunity) in the face of adverse events. These factors include personal control, positive affect, social support, and optimism. Although there is limited evidence here for these resilience factors protecting persons from stress-induced immune changes, evidence on effects on psychological well-being, and autonomic response provides indirect support for these hypotheses. It is important to note that other factors such as greater self-efficacy, purpose in life, and self-esteem have all been associated with psychological resilience in the face of stressful life events see Bonanno et al. (2011) and Adler and Matthews (1994), although they all lack a sufficient evidence base in regard to their implications for stress-induced changes in biological responses, including immunity.

It should be noted that the evidence we review here is based on studies of the effectiveness of individual resilience factors. In other words, this literature does not address the possibility that some factors may be correlated, and actually represent the same underlying concept. We emphasize studies that focus explicitly on resilience, sometimes called stress-buffering. Such studies include measures (or manipulations) of both a stressor and the resilience factor under consideration and predict that the stressor will be related to a deleterious health outcome for those low in resilience, but not for those high in resilience (stress-by-resilience factor interaction).

**Fig. 1:**
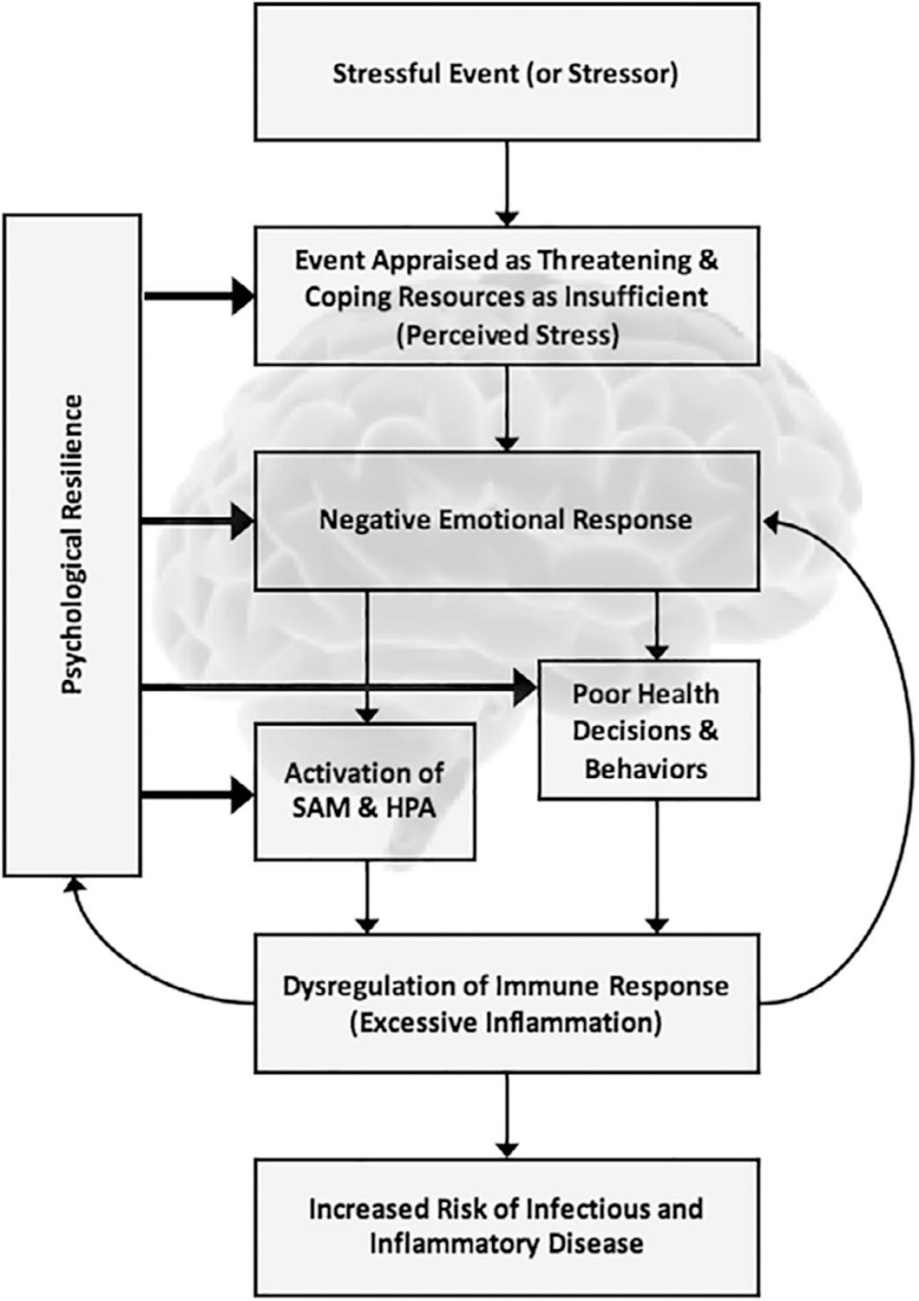
A heuristic model of how stressful life events can influence immunity and health (downward arrows), the potential feedback of excessive inflammation on the process (curved arrows), and the points at which psychological resilience factors can short-circuit this process (horizontal arrows). Adapted from Cohen et al. (2016).

Stress is thought to influence health both by promoting behavioral coping responses detrimental to health (smoking, drinking alcohol, illicit drug use, sleep loss) and by activating physiological systems that support behaviors required to cope with stressors such as the sympathetic nervous system (also called the sympathetic adreno-medullary (SAM) system) and the hypothalamic-pituitary-adrenal (HPA) axis (Cohen et al., 1995; McEwen, 1998). Prolonged or repeated activation of these systems is thought to place persons at risk for the development of a range of physical (e.g., immune, cardiovascular, metabolic) and psychiatric disorders. In general, psychological resilience is thought to operate by attenuating the appraisal of the threat posed by a stressor, by providing effective means of coping with stressors that are appraised as threatening, and by reducing affective (e.g., anxiety, depression, anger), behavioral (e.g., smoking, alcohol consumption, loss of sleep, poor diet), and in turn physiological (e.g., activation of SAM and HPA systems) responses to stressors that put people at risk for immune dysfunction and consequently for infectious and inflammatory diseases (Cohen et al., 2016) (Fig. 1).

#### 3.2.1. Personal control

One factor conceptually and empirically linked to resilience is high perceived control, or, beliefs in one’s ability to influence circumstances and attain goals (Benight and Bandura, 2004; Dunkel Schetter and Dolbier, 2011). Conceptual models of stress, coping, and health (Lazarus and Folkman, 1984; Pearlin et al., 1981) posit that greater perceived control may reduce the perception of threat when appraising stressful events, as well as promote more adaptive coping responses (e.g., problem-solving, support-seeking). This may, in turn, reduce the severity or chronicity of negative cognitive and emotional states, as well as stress-related physiological alterations, evoked by potentially stressful experiences.

There is considerable evidence that feelings of personal control provide protection from the negative emotional responses associated with major stressful life events as reviewed by Cohen and Edwards (1989). There are also many experimental studies where control over laboratory stressors reduced or totally ameliorated stress-induced increases in sympathetic activation (heart rate, blood pressure, galvanic skin response). These include studies where subjects actually engage in a behavior that controls the onset, duration, or intensity of a stressor e.g., Geer et al. (1970) and Glass (1973). However, it also includes situations where the mere perception that control is possible is an effective stress-buffer (Glass, 1972).

Field studies have similarly found that among those with high perceived control there is an attenuation of the associations of major stressful life events with negative affect and depressive symptoms e.g., King et al. (2015) and Mausbach (2007); of the association of lifetime trauma and the inflammatory marker, CRP (Elliot et al., 2017); and the associations between major depression and poorer natural killer cell activity (NKCA) (Reynaert, 1995). In a recent study, Elliot et al. (2018) found that perceived control also protected against the increase in allcause mortality associated with a greater number of lifetime traumatic experiences even with adjustments for baseline health status, and psychological, and behavioral covariates.

#### 3.2.2. Positive affect

Positive affect (PA) includes the feelings that reflect a level of pleasurable engagement with the environment (Clark et al., 1989) such as happiness, joy, excitement, enthusiasm, and contentment. Although these resources may be short-lived, they may also be long lasting and may be drawn upon in moments of need. The “broaden and build” theory (Fredrickson, 1998) argues that PA acts as a resilience factor by encouraging exploration and creativity resulting in the building of social, intellectual, and physical resources via interactions and exploration (e.g., juvenile play) by broadening action tendencies. Other theories of why PA would buffer the effects of stress include the possibility that positive emotions generate creative problem solving (Ashby et al., 1999), encourage restorative activities such as sleep, exercise, relaxation, vacation, and spending time in natural environments (Smith and Baum, 2003) or protect persons from negative responses to stress through the release of endogenous opioids that diminish autonomic and endocrine responses that are often triggered by stress (Smith and Baum, 2003).

There is no doubt that positive affect and subjective well-being are associated with better self-reported health, lower morbidity, less pain, and longevity, see reviews by Diener and Chan (2011), Lyubomirsky et al. (2005), Pressman and Cohen (2005), Veenhoven (2008), Chida and Steptoe (2008) and Howell et al. (2007). An analysis across 142 nations found that positive emotions predict better self-rated health around the world, with positive emotion trumping hunger, shelter, and safety in predictive value (Pressman et al., 2013).

There is also increasing evidence that PA facilitates recovery from stress-related activation. To date, studies have found that inducing amusement or contentment following a stressful or fearful stimulus results in a faster return to baseline levels of cardiovascular reactivity as does spontaneous smiling during a sadness-inducing stimulus (Fredrickson and Levenson, 1998; Fredrickson et al., 2000). In line with this, ambulatory studies have shown that heart rate increases last a shorter period after positive versus negative affect (Brosschot and Thayer, 2003) and that high happiness occurring naturally during a period of high anxiety counteracts blood pressure increases that occur in the absence of happiness (Shapiro et al., 2001). Similarly, there is some evidence of PA’s buffering the negative immune impact of ambulatory negative mood (Valdimarsdottir and Bovbjerg, 1997). Here higher levels of positive mood were related to higher levels of natural killer cell activity only among the women who reported having some negative mood over the day. These results raise the possibility that positive mood is protective against the effects of negative mood on immune function.

#### 3.2.3. Social support

Social support refers to a social network’s provision of psychological and material resources intended to benefit an individual’s ability to cope with stress (Cohen, 2004). It is often differentiated in terms of three types of resources: instrumental, informational, and emotional (House et al., 1985). Instrumental support involves the provision of material aid, for example, financial assistance or help with daily tasks. Informational support refers to the provision of relevant information intended to help the individual cope with current difficulties and typically takes the form of advice or guidance in dealing with one’s problems. Emotional support involves the expression of empathy, caring, reassurance, and trust and provides opportunities for emotional expression and venting. Such typologies of support provide a basis for determining whether the effectiveness of different kinds of support differs by the nature of stressful events or by the characteristics of persons suffering adversity.

The current literature suggests that the critical factor in social support operating as a stress buffer is the perception that others (even one reliable source) will provide appropriate aid (Cohen and Wills, 1985; Uchino et al., 1996; Cohen, 1988). In this view, the belief that others will provide necessary resources may bolster one’s perceived ability to cope with demands, thus changing the appraisal of the situation and lowering its effective stress (Cohen and Wills, 1985; Thoits, 1986; Wethington and Kessler, 1986). Belief that support is at hand may also dampen the emotional and physiological responses to the event or reduce maladaptive behavioral responses e.g., (Wills and Cleary, 1996).

There is substantial evidence that the perceived availability of social support buffers the effect of stressors on psychological distress, depression, and anxiety, reviewed by Cohen and Wills (1985) and Kawachi and Berkman (2001). For example, both student and adult samples report more symptoms of depression and of physical ailments under stress but these associations are attenuated among those who perceive that support is available from their social networks (Cohen, 2004). When types of perceived support were broken down, emotional support acted as resilience factors in the face of a variety of types of stressful events, whereas other types of support (e.g., instrumental, informational) responded to specific needs elicited by an event.

The most striking evidence for stress buffering in the physical health realm is reported in a prospective study of healthy Swedish men aged 50 years and over (Rosengren et al., 1993). Those with high numbers of stressful life events in the year before the baseline exam were at substantially greater risk for mortality over a seven-year follow-up period. However, this effect was ameliorated among those who perceived that high levels of emotional support were available to them. In contrast, perceived emotional support made no difference for those with few stressful events.

A recent study (Cohen et al., 2015) similarly found that participants who had multiple social conflicts in their lives were at greater risk of developing an infection when subsequently exposed to a common cold virus than those with fewer or no conflicts. However, the association of greater conflict and increased risk of infection held only for those who reported that they expected little support from others when they face aversive events. In short, those with greater perceived support were protected from stress-associated susceptibility to infection.

Beyond perceptions, the actual receipt of support could also play a role in stress buffering. Support may alleviate the impact of stress by providing a solution to the problem, by reducing the perceived importance of the problem, or by providing a distraction from the problem. It might also facilitate healthful behaviors such as exercise, personal hygiene, proper nutrition, and rest, cf. (Cohen, 1988; House, 1981). Work with primates has shown that those that spend more of their time engaged in affiliative behaviors (e.g., touch, close proximity) showed less suppression of mitogen stimulated lymphocyte proliferation following a continuous exposure to a social stressor (living in an unstable as opposed to stable social group) than those spending less time affiliating with other animals (Cohen et al., 1992). Interestingly, humans who reported being regularly hugged by others showed a similar resilience to the potential immune effects of conflicts with others. In a viral-challenge study, those with conflict but few days being hugged were at greater risk of infection in response to exposure to a common cold virus, but those who received many days of hugging were protected from the role of conflict in increasing risk (Cohen et al., 2015).

#### 3.2.4. Optimism

Dispositional optimism reflects the extent to which individuals hold generalized favorable expectancies for their future, and pessimism reflects the extent to which they hold unfavorable expectancies (Carver et al., 2010). Optimism has been consistently associated with better psychological adjustment and self-reported physical health in response to diverse life transitions including entering college, pregnancy, cardiac surgery, and caregiving, see Carver and Scheier (1999), for review. However, studies of biological outcomes provide a less clear picture of the role of optimism as a resilience factor in the face of stressful events (Segerstrom, 2005). For example, while in one study, those with higher levels of optimism were found to have substantially lower risk of rehospitalization following coronary artery bypass surgery (Scheier et al., 1999); another study found no relationship between optimism, recovery and length of stay following cardiac surgery (Contrada et al., 2004). Studies of HIV and cancer patients show similar inconsistencies in results, see review by Segerstrom (2005).

Studies of optimism as a potential buffer of the effects of stressors on immune function also show mixed outcomes, see reviews by Segerstrom (2005) and Cohen et al. (2012). A recent review by Cohen et al. (2012), attributes the inconsistencies to insufficient samples sizes (Ns ranging from 22 to 59) to provide the statistical power required to test the predicted stressor-by-optimism interactions that would provide support for the resilience hypothesis. In addition, these studies are based primarily on trait measures of optimism, when measures assessing optimism in relation to the specific situation would, in theory, be a more effective in buffering the potential effects of a stressor. In contrast, Segerstrom (2005) hypothesizes that optimism is associated with less cellular immunity when stressors are complex, consistent or un-controllable, but positively related when stressors are straight forward, brief and controllable and that these differences are attributable to optimists’ greater engagement during difficult stressors. Either way, what is clear is that the existing literature is insufficient in either number of studies or consistency in results to establish optimism as a buffer of the effects of stressors on immunity and physical disease outcomes.

### 3.3. Issues in interpreting the literature on the influence of resilience factors on immunity

Most of the human research presented above is based on the potential role of trait characteristics as resilience factors. We do not interpret this literature as evidence for traits being responsible (or required) in order to achieve resilience. Rather we assume that a resilience factor represented by a trait (e.g., personal control, or optimism) needs to be engaged in response to adverse situations in order to contribute to the resilience process, for example to result in reinterpretation of a stressor or the choice of a successful coping strategy. Persons with specific traits (e.g., those high in control/mastery, optimism, or perceptions of social support) are most likely to engage the represented factor successfully in response to a specific challenge and most likely to engage it successfully in response to an experimental manipulation of the corresponding state. However, a state manipulation of the factor, in even low trait individuals, can be successful to the extent it triggers relevant downstream processes.

The interpretation of this literature is severely limited by the cross-sectional correlational design used in many of the studies. In this design, assessments of all variables (stressors, proposed resilience factors, and physiological outcomes) are assessed concurrently. Consequently, causal inference is not possible, and the results may be attributable to stress resulting in immune change, or alternatively to immune change resulting in stress. Many of these correlational studies also lack inclusion of controls for third (spurious) factors such as age, socioeconomic status, or race/ethnicity that may be the actual causal factors resulting in changes in the resilience factors and the outcome. It is noteworthy that these are not inherent problems in the study of humans and these issues could be addressed in experimental studies or studies using prospective longitudinal designs where the stressor and proposed resilience factors are used to predict changes in immunity over time and appropriate controls (covariates) are included.

## 4. Immune influences on resilience

To better understand how the immune system modulates the resilience process we will first describe the mechanisms by which peripherally occurring immune responses modify brain functions and can ultimately result in profound changes in behavioral priorities. Although these immune influences on the brain are usually reversible they can also lead to pathology and increase the risk for developing psychiatric disorders such as major depressive disorder. The mechanisms that are involved are the same as those that modulate immune influences on resilience. Among other examples of these influences we will examine in particular how individual differences in the innate immune system can impair resilience in face of social stressors by compromising integrity of the blood-brain barrier. The data we discuss in this section should not be interpreted to mean the immune system is always driving how we cope with adverse events or the way we navigate our social environment. What happens in situations of activation of the immune system triggered by pathogen-associated molecular patterns or by potentially injurious stressors does not necessarily generalize to other circumstances in which cytokine levels fluctuate in response to e.g., exercise (Huh, 2018) or pharmacological treatment (Zeiser, 2018).

### 4.1. Immune influences on brain functions

As discussed in the previous section, there is increasing evidence from human studies that psychosocial factors intervening in the resilience process modulate immune system functions. These influences take place mostly via neuroendocrine and neurohormonal stress pathways. However, the relationship between the central nervous system and the immune system is not unidirectional, from the brain to the immune system, but fully bidirectional, from the immune system to the brain (Dantzer, 2018). Both human and animal studies show that immune mediators are able to influence the way the brain processes information and responds to it both physiologically and behaviorally (Dantzer et al., 2008). The best example of this influence of the immune system on brain function is represented by the profound adjustments in physiology and behavior that take place during an inflammatory process. Acute activation of the innate immune response by pathogen-associated molecular patterns recognized by leukocytic toll-like receptors and inflammasome triggers an adaptive response mediated by the interplay between proinflammatory and anti-inflammatory cytokines. Proinflammatory cytokines are produced *de novo* by activated macrophages. They coordinate the different facets of the inflammatory response in the environment in which the innate immune system is mobilized, and they allow the development of the adaptive immune response to facilitate the response to further challenge from the same antigens. They also act at distance to trigger the acute phase reaction that includes recruitment of hepatocytes to produce acute phase proteins, increase in the hypothalamic thermoregulatory set point to set up the fever response, and induction of behavioral and neuroendocrine responses to facilitate the metabolic adjustments made necessary by the energy requirements of the fever response. The most obvious aspect of the necessary reorganization of priorities that takes place in an organism having to cope with an infection is the episode of sickness behavior it displays, in the form of reduced locomotor activity, decreased interactions with the physical and social environment, reduced appetite, and curling posture to decrease heat losses. This is made possible by several immune-to-brain communication pathways involving neural pathways (the afferent nerves that innervate the site of inflammation) and humoral pathways (e.g., the circulating pathogen-associated molecular patterns that act on macrophages-like cells in the circumventricular areas and the choroid plexus that are devoid of a blood-brain barrier and trigger there the local production of cytokines that then propagate into the brain via volume transmission) (Konsman et al., 2002). The production and actions of proinflammatory cytokines are regulated by a number of opposing mechanisms involving anti-inflammatory cytokines such as IL-10, glucocorticoids, and several neuropeptides including vasopressin. There is evidence that the neuroimmune mechanisms that organize sickness behavior can also be recruited by non-immune stressors including psychosocial life events (Marsland et al., 2017; Michopoulos et al., 2017; Frank et al., 2015). In addition, immune and non-immune stressors occurring in the perinatal period have long-term effects that persist in adulthood and shape the way the immune system and the brain will respond to environmental challenges (Meyer, 2014; Weir et al., 2015; Bilbo et al., 2018; Brown and Patterson, 2011).

The mechanisms that are involved in the interactions between the central nervous and the immune systems have already been described in details (Dantzer, 2018). The purpose of the present review is not to describe once more these mechanisms but to present what is specific to the resilience process. We will show that the essential components of the resilience process are modulated in quality and intensity by precisely those immune molecules that mediate the response to immune and non-immune stressors.

### 4.2. From sickness to symptoms of depression

Although acute inflammation predominantly induces feelings of sickness, prolonged inflammation can lead to depression in the presence of risk factors for depression such as early adversity, lack of social support, or single nucleotide polymorphism in cytokine genes resulting in increased expression of inflammatory cytokines or decreased expression of anti-inflammatory cytokines (Dantzer and Capuron, 2017; Raison and Miller, 2011). Inflammation-induced depression differs from major depressive disorder by the predominance of somatic symptoms such as reduced appetite, sleep disorders and fatigue in affected individuals. However, the cognitive and affective symptoms of depression can also be present in 20–40% of the affected individuals and they usually develop later than somatic symptoms. This dissociation between the somatic and cognitive/affective symptoms of depression is also apparent when patients are treated with antidepressant drugs. Administration of specific serotonin reuptake inhibitors such as paroxetine prevents the development of the affective and cognitive dimensions of depression in inflamed patients but is less effective on somatic symptoms (Capuron et al., 2002).

**Fig. 2:**
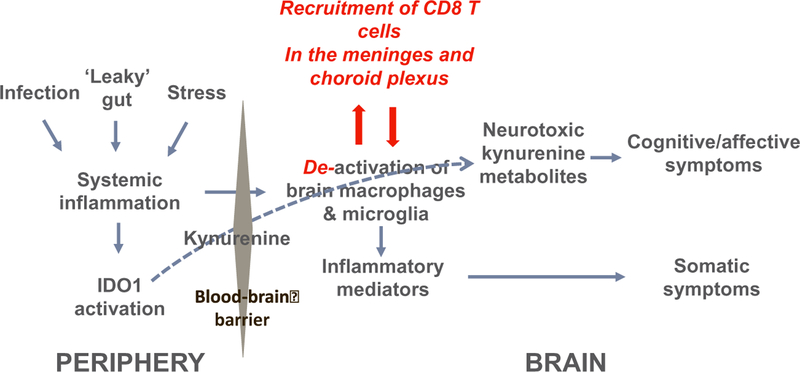
Mechanisms of inflammation-induced depression and its recovery. Systemic inflammation in response to infection, ‘leaky’ gut or stress activates indoleamine 2,3 dioxygenase (IDO1) and induces the expression of inflammatory mediators by brain macrophages and microglia. Activation of IDO1 leads to the increased formation of kynurenine. Circulating kynurenine is transported through the blood-brain barrier and converted into neurotoxic kynurenine metabolites by enzymes mainly expressed in microglia. Somatic symptoms of depression are the result of the action of brain inflammatory mediators on neuronal networks regulating arousal and incentive motivation. Cognitve/affective symptoms of depression are mediated in part by the action of neurotoxic kynurenine metabolites on glutamatergic neurotransmission. Activation of brain macrophages and microglia recruits CD8-positive T cells into the meninges and choroid plexus. These T cells switch brain macrophages and microglia into an anti-inflammatory phenotype that is pivotal for the recovery process. Note the blue color for the mechanism that lead from inflammation to depression and the red color for the mechanisms that promote recovery. (For interpretation of the references to color in this figure legend, the reader is referred to the web version of this article.)

Based on the results of clinical and preclinical studies, it has been proposed that the switch from sickness to the cognitive/affective symptoms of depression is mediated by immune-dependent activation of the tryptophan metabolizing enzyme, indoleamine 2,3 dioxygenase (IDO1) (Dantzer et al., 2008) (Fig. 2). IDO1 is activated by cytokines such as interferon-gamma and tumor necrosis factor. It converts the essential amino acid tryptophan to N-formyl-kynurenine that is further metabolized to kynurenine. Activation of IDO1 and increased formation of kynurenine play an important role in the physiological regulation of the immune response as kynurenine itself increases the production of regulatory T-cells by activating the aryl hydrocarbon receptor (Mezrich et al., 2010). However, kynurenine produced by activated macrophages and dendritic cells acts not only at the periphery but also in the brain where it is transported by the same transporter that regulates the entry of tryptophan and other neutral amino acids in the brain (Dantzer, 2017). In the brain, kynurenine is further enzymatically metabolized into neuroprotective metabolites such as kynurenic acid, and into neurotoxic metabolites such as 3-hydroxy kynurenine and quinolinic acid. During inflammation, more kynurenine enters the brain and the neurotoxic branch of the kynurenine metabolism pathway predominates over the neuroprotective branch. This results in increased excitotoxicity as quinolinic acid is a potent agonist of the N-methyl D-aspartate receptor. In addition, proinflammatory cytokines decrease dopaminergic neurotransmission by reducing the bioavailability of tetrahydrobiopterin, a cofactor of hydroxylase enzymes such as phenylalanine hydroxylase that converts phenyl alanine to tyrosine and tyrosine hydroxylase that converts tyrosine to dihydroxyphenyl acetic acid (Strasser et al., 2017). Tryptophan hydroxylase is also affected, which leads to reduced synthesis of serotonin. Proinflammatory cytokines also increase extracellular glutamate by facilitating the release of glutamate from microglial cells and disrupting the glutamate-glutamine cycle in astrocytes. All these effects converge on reduced monoaminergic neurotransmission and increased glutamatergic neuro-transmission (Felger and Treadway, 2017; Haroon et al., 2017). Non-immune stressors have the same effect although part of the increased production of kynurenine is probably mediated by the activating effects of cortisol on hepatic tryptophan 2,3 dioxygenase (TDO2) (Gibney et al., 2014).

### 4.3. Evidence for immunity modulating psychological resilience

In support of the potential influence of immunity on psychological resilience in humans, there is considerable evidence from cross-sectional correlational studies for positive affect, optimism, control, and social support being associated with a broad range of immune measures, see review for instance in Marsland et al. (2017), Cohen et al. (2012) and Uchino et al. (1996). Although consistent with immunity modulating psychological resilience factors, these data are limited in that the direction of causality is unclear, and it is just as possible (and as likely) that reported associations are attributable to psychological states influencing immunity as it is that immunity contributes to psychological states.

More compelling evidence for the role of immunity in psychological states contributing to resilience is the literature (see Section 4.1) showing that chronic inflammation and infectious diseases commonly trigger nonspecific psychological and behavioral changes including fatigue and malaise, anhedonia, inability to concentrate, social withdrawal and disturbed sleep that collectively are termed “sickness behaviors” (Dantzer et al., 2007). Converging evidence from several lines of research implicate the activities of pro-inflammatory cytokines as a cause of sickness behaviors (Dantzer et al., 2008). In addition to the behaviors mentioned above, emotional responses are thought to be more generally influenced by infectious disease and consequent inflammatory responses. Here we pay specific attention to the possibility that two of the resilience factors discussed in Section 3, positive affect and social support, may be regulated by the immune systems release of pro-inflammatory cytokines.

Although a broad range of studies have indicated a role of pro-inflammatory cytokines in increasing levels of anxiety and depressed mood e.g., (Irwin and Miller, 2007; Reichenberg, 2001), the evidence in relation to immune effects on positive affect is limited. This includes two studies that have examined the relation of cytokine release to changes in positive affect following exposure to mild pathogenic stimuli. In the first (Wright et al., 2005), administration of S. typhi vaccine, but not placebo, was associated with increased IL-6 concentrations, increased negative mood, and decreased positive mood within the first three hours post-injection. Post-injection negative mood, but not positive mood, was correlated with an increase in IL-6. In the second (Janicki-Deverts et al., 2007), healthy adults were experimentally exposed to rhinovirus (RV) or influenza virus (FLU) followed by a 5 (RV) or 6 (FLU) day period of quarantine. Infection, objective signs of illness, nasal IL-1β, IL-6, and TNF-α, and self-reported affect were assessed at baseline and on each of the post-challenge quarantine days. In the 153 persons who became infected following exposure to the challenge virus, daily production of IL-6, but not IL-1β or TNF-α, was associated with reduced concurrent (same day) daily positive affect. One-day lagged prospective analyses showed that daily production of all 3 cytokines was related to lower positive affect on the next day. All lagged associations were independent of previous day positive affect and objective signs of illness (mucus production, mucociliary clearance function). There were no associations between cytokines and next day negative affect. Findings support a causal association (cytokines predict the change from one day to the next) between infection-induced local cytokine production and decreases in positive affect over the following day.

In short, these studies provide preliminary evidence for a decrease in positive mood in response to mild pathogenic stimuli. Moreover, although the data are mixed (possibly due to Wright et al. only assessing one inflammatory marker, IL-6 (Wright et al., 2005)), there is some indication that this association is mediated through inflammatory response.

Evidence that activation of the innate immune system modifies the way an individual navigates the social environment is summarized in a review of both animal and human research by Eisenberger et al. (2017). They conclude that inflammatory processes regulate social behavior, leading to characteristic changes that may help an individual navigate the social environment during times of sickness. This includes increases in threat-related neural sensitivity to negative social experiences (e.g., rejection, negative social feedback), and enhanced reward-related neural sensitivity to positive social experiences (eg, viewing close others and receiving positive social feedback). For example, in one study, participants exposed to a low dose of endotoxin, a safe trigger of inflammation, (versus placebo) reported greater desire to be with their close others during the peak inflammatory response (Inagaki et al., 2015). Another showed that that participants exposed to endotoxin (versus placebo) displayed greater reward related activity in response to receiving positive feedback; and heightened neural activity in a number of threat-related neural regions in response to negative feed-back from an evaluator (Muscatell et al., 2016). This work with humans along with research in animals, indicates the important role of inflammation in social behaviors that have implications for resilience.

### 4.4. Resilience to stress-induced depression: role of individual differences in the immune system

Resilience, defined by the ability to successfully adapt in the face of severe stress, may reflect individual differences in behavioral coping strategies (Russo et al., 2012). Indeed, rodent and human studies of resilience indicate that active versus passive coping strategies are protective and help to overcome adversity and resist the development of stress-induced psychiatric disorders (Russo et al., 2012; Charney, 2004; Feder et al., 2009). As described by Koolhaas et al. (1999), Koolhaas (2008) and Koolhaas et al. (2007), active coping individuals from an outbred strain of rats take more initiative and exhibit characteristics such as aggression, active avoidance, and defensive burying, when confronted with a potential adverse situation, whereas passive coping individuals seem to accept their circumstances and act only when they absolutely have to. While much of the human literature—by necessity—has focused largely on neuroendocrine alterations associated with active coping or resilience, animal studies are beginning to provide causal evidence of active adaptive processes in the brain or periphery that promote resilience (Russo et al., 2012).

Repeated social defeat is a well-established model of chronic stress and can be employed in mice or rats to study resilience or susceptibility to depression-like behavior (Golden et al., 2011; Wood et al., 2010). In this model, individual rats or mice are repeatedly confronted to an aggressive conspecific for a few minutes before being separated and in some cases forced to cohabit with the aggressor separated from them by a wired barrier. This procedure results in the development of many of behavioral signs of depression, including decreased sucrose preference, social avoidance, body weight loss, and disruption of circadian rhythms. Resilient individuals typically show very little of these behavioral signs of depression. Resilience to developing depression-like behavioral phenotypes following exposure to social defeat stress is associated with individual differences in the immune system (Hodes et al., 2014; Wood et al., 2015). Though it is not yet fully clear whether resilience is the cause or consequence of these individual differences in the immune system, emerging evidence does suggest that it should be possible to reverse the deleterious consequences of passive coping to stress by reducing stress-induced inflammation. For example, Wood et al. (2015) examined inflammatory cytokines in the locus coeruleus (LC) and dorsal raphe (DR) of the brain of passive versus active coping rats in the social defeat stress model. They found that compared to active coping rats, passive coping rats assumed a subordinate behavioral response to a dominant rat, developed anhedonia measured by decreased preference for a sucrose solution over water, and exhibited elevated cytokine expression in the LC and DR. Interestingly administration of the type I IL-1 receptor antagonist into the lateral ventricle of the brain before each daily social defeat session reduced anhedonia in the passive coping rats. In addition, Hodes et al. (2014) found that there are preexisting individual differences in the peripheral immune system that promote susceptibility versus resilience to social defeat stress in mice. In particular, they observed that resilient mice had lower circulating levels of the pro-inflammatory cytokine IL-6 in response to acute stress than their susceptible counterparts. The same difference was found for IL-6 produced by peripheral blood mononuclear cells stimulated *in vitro* by a standard dose of the cytokine inducer lipopolysaccharide (LPS). Sequestration of peripheral IL-6 with neutralizing antibodies or generation of chimeric mice that lack IL-6 in their bone marrow-derived leukocytes promoted resilience to stress. When taken together, these studies highlight the fact that differences in both peripheral and central inflammatory processes are causally linked to stress susceptibility, raising the exciting possibility of targeting inflammation to promote resilience through active behavioral coping.

#### 4.4.1. Targeting neurovascular regions to promote resilience

**Fig. 3:**
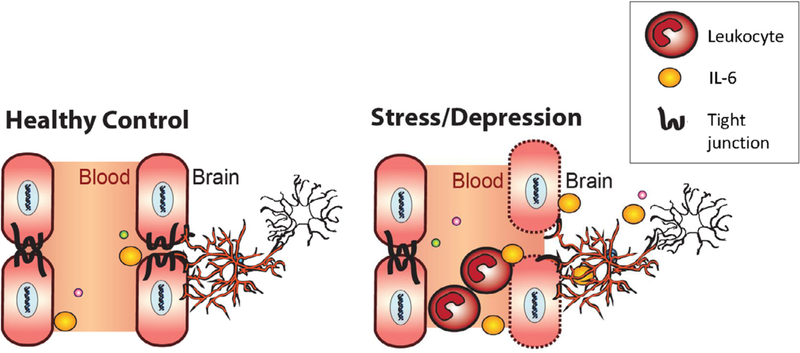
Schematic depicting effects of chronic stress on endothelial barrier. Chronic social defeat stress in mice and depression in humans is associated with increased circulating levels of leukocytes (largely monocytes and neutrophils) and reduced expression of the tight junction protein claudin-5 (CLDN5). In mice loss of CLDN5 results in an opening of the blood brain barrier that allows circulating inter-leukin-6 (IL-6) to directly enter the brain where it might act on astrocytes (depicted by red cells in the brain) or microglia (depicted by white cells in the brain) within mood related structures such as the nucleus accumbens to increase depression-like behaviors compared to resilient and control mice. Although, untested, there may be alterations in astrocytes or microglia induced by peripheral IL-6 infiltration that further promote blood-brain barrier permeability and/or neuroinflammation. Adapted with permission from Menard et al. (2017).

Based on the preclinical studies described above, it has been hypothesized that circulating inflammatory molecules released in response to chronic stress exposure, penetrate the blood brain barrier (BBB), and affect neural circuits that mediate stress vulnerability and depression. Thus, it is possible that resilience versus susceptibility depends on neurovascular health and maintenance of the integrity of the BBB to prevent stress-induced inflammatory molecules from entering mood-related brain structures. The BBB is comprised of multiple protective layers including endothelial cells and astrocytes, which play critical roles in maintaining vascular impermeability (Neuwelt et al., 2011). Endothelial cells, via expression of tight junction proteins, establish the paracellular barrier between the blood and perivascular space. Astrocytes provide a secondary barrier between the perivascular space and the brain parenchyma. As shown in Fig. 3, breakdown of the endothelial barrier or loss of astrocyte function and/or density can lead to infiltration of peripheral immune signals—such as IL-6—that have been shown previously to increase stress susceptibility measured by social avoidance following the social stress test (Hodes et al., 2015; Menard et al., 2017).

Much attention has been paid in the past to the strong link between stress and BBB permeability. Both acute and chronic stress in rodents are able to increase permeability to a range of peripherally administered dyes of varying sizes from ∼0.5 kD all the way up to ∼70 kD (Friedman et al., 1996; Menard et al., 2017; Santha et al., 2015; Sharma and Dey, 1981; Esposito et al., 2001). Increased permeability of the BBB has also been suggested to occur in human patients with stress-related disorders, such as depression (Niklasson and Agren, 1984). However, causal links between BBB permeability and depression-like behavior were only recently established (Menard et al., 2017). Utilizing the model of chronic social defeat stress described above, it was found that stress selectively affects endothelial tight junctions of susceptible, but not resilient mice, in the nucleus accumbens (NAc), a brain region integrally involved in depression symptomatology. Following just 10 days of repeated social defeat, claudin 5 (Cldn5) mRNA and protein expression were reduced in the NAc of stress-susceptible mice that exhibited depression-related behavioral phenotypes when compared to resilient mice and unstressed controls. A similar decrease of Cldn5 mRNA was also observed in the NAc of depressed patients. Interestingly, chronic treatment of mice with the antidepressant imipramine promoted resilience and rescued Cldn5 expression in the NAc. Moreover, chronic down-regulation of Cldn5 expression with an adeno-associated virus including a short hairpin RNA specific to Cldn5 was sufficient to induce social avoidance and depression-like behaviors as assessed by decreased sucrose preference and increased immobility in the forced swim test. It was further established that reduced Cldn5 expression in NAc promoted greater BBB permeability and peripheral infiltration of peripheral molecules, including the proinflammatory cytokine IL-6. Interestingly this study showed that blood-brain permeability was not brain-wide but largely restricted to certain mood related regions. One possibility is that stress-induced neuronal activity in these structures might lead to local production of damage signals via the recruitment of local microglia. In addition, a recent study indicated that local microglia are necessary for recruitment of bone marrow-derived monocytes to the neurovasculature (McKim et al., 2017). Although more work is needed to clarify this point, region specific recruitment of these inflammatory monocytes by microglia might promote a local inflammatory signal that promotes localized endothelial damage.

Additional studies have also linked possible vascular damage to stress vulnerability. First, Pearson-Leary et al. (2017) found greater vascular remodeling in the hippocampus of passive coping rats compared to active coping rats. This difference was attenuated by systemic administration of meloxicam, a non-steroidal anti-inflammatory drug. However, in this study it was not determined whether the vascular remodeling was sufficient to alter BBB permeability and peripheral immune infiltration. More recently, Cheng et al. (2018) found that depressive behavior of mice in the learned helplessness model of depression was associated with increased BBB permeability. This effect could be reversed by administration of either the TNF inhibitor etanercept, or TDZD-8, an inhibitor of glycogen synthase kinase (GSK). TNF and GSK are being actively investigated as new therapeutics targets in human depression as well (Costemale-Lacoste et al., 2016; Raison, 2013). Together, these findings highlight a protective role for the BBB against the development of depression-like behaviors in situations of stress. By understanding how chronic stress affects the BBB, we may be able to augment current antidepressant treatment or design new therapeutic strategies promoting vascular health and resilience by preventing BBB leakage.

#### 4.4.2. Role of the immune system in the recovery process

In the resilience literature, resilience is sometimes opposed to recovery. This is done by referring to resilience as the ability to maintain normal physiological and mental functioning in face of adversity and to recovery as a distinct process that operates to restore normality when exposure to adverse conditions has altered the functioning of the organism (Bonanno, 2004). Our idea of the resilience process is more inclusive as it includes both the capacity to adapt to adverse conditions, as described in the previous section, and the ability to recover from the strain imposed by the adaptation process on behavior and physiology. The immune factors that are involved in the recovery process have been much less studied than those that are responsible for the ability to sustain adversity and there is no reason a priori for them to be the same. Mechanistic differences between the two components of the resilience process, resist versus bounce back, have been identified in a series of recent studies on the recovery from inflammation-induced pain and depression. In these studies, inflammation is the triggering factor for the adverse condition to which individuals must adapt. It induces an episode of pain and depression from which inflamed individuals emerge after a few days or weeks. The ability to resist the development of pain and depression is mediated by innate immunity. However, the recovery process is dependent on the involvement of the adaptive immune system.

When mice are treated with the chemotherapeutic agent paclitaxel, they experience pain from which they normally recover within 1 week after treatment. The development of pain involves inflammatory mechanisms as it can be prevented by anti-inflammatory agents. T cell deficient mice also develop pain in response to paclitaxel but they take longer to recover as they display prolonged hypersensitivity to mechanical stimuli for at least 3 weeks after treatment (Krukowski et al., 2016). The duration of this abnormally long mechanical allodynia was normalized by adoptive transfer of T cells, and more specifically CD8 T cells from normal mice (Fig. 2). The specific genetic mutant mice used in these experiments were mice genetically deficient in *rag1*, a recombinant activation gene that is necessary for B and T cell differentiation. CD8 T cells were found to be present at the time of recovery in the lumbar dorsal root ganglia of wild type but not *rag1*−/− mice. As CD8 T cells exert their regulatory effects on paclitaxel-induced inflammation by the production of the anti-inflammatory cytokine IL-10, the next step in these experiments consisted of neutralizing IL-10 by intrathecal injection of a neutralizing antibody to IL-10. Administration of this treatment at the normal time of recovery delayed recovery in wild type mice. The same effect was apparent in *rag1*−/− mice reconstituted with CD8 T cells from normal mice. The importance of IL-10 for promoting recovery was apparent from the absence of recovery in *il-10*−/− mice treated by paclitaxel.

To determine whether the same mechanism applies to recovery from inflammation-induced depression, similar experiments were conducted in *rag2*−/− mice compared to wild type mice and treated with lipopolysaccharide to induce depression-like behavior (Laumet et al., 2018). *Rag2*−/− mice were preferred to *rag1*−/− mice as *rag2* has the same function in the immune system but in contrast to *rag1* it is not expressed in the central nervous system. The experiments carried out on the depression-like behavior of lipopolysaccharide-treated *rag2*−/− mice provided essentially the same results as those obtained in paclitaxel-treated mice. *Rag2*−/− mice took longer to recover from lipopolysaccharide-induced depression than wild type mice, and this was associated to a sustained increased expression of IDO1 in their brains. This deficit was abrogated by reconstitution of their immune system with CD8 T cells. CD8 T cells were found to infiltrate the meninges and choroid plexus of lipopolysaccharide-treated wild type mice at the time of recovery. IL-10 was also found to be a critical factor as intranasal administration of a neutralizing antibody to IL-10 to target the meninges prolonged lipopolysaccharide-induced depression-like behavior in wild type mice.

These experiments are important as they demonstrate that recovery from inflammation-induced depression-like behavior is an active process that requires an intact adaptive immune system. CD8 T cells most likely induce the production of IL-10 by resident macrophages and microglia. This anti-inflammatory cytokine down regulates macrophage and microglia activation in the central nervous system. Whether such a process mediates individual differences in the ability to bounce back from other stressors and in particular non-immune stressors is unknown.

#### 4.4.3. Promoting resilience through vaccination (or inoculation) to stress.

One important area of active research into resilience and the immune system centers on the idea that it could be possible to inoculate an individual against repeated stress much like how individuals are inoculated against viral pathogens through vaccines. For example, it was shown that vaccination with a weak, central nervous system (CNS)-specific, myelin-derived peptide agonist prevented emergence of depression-like behaviors following chronic mild stress in rats (Lewitus et al., 2009). Similarly, T cell–deficient mice showed enhanced vulnerability to stress, whereas mice overexpressing auto-reactive T cells were resistant to depression-like behavior when confronted with a predator odor challenge (Cohen et al., 2006). Interestingly, T-cell therapy replacement strategies in T cell–deficient mice promoted resilience to stress. A more recent study showed that transplantation of lymph node cell suspensions from previously stressed mice produced antidepressant effects and reduced circulating inflammatory cytokine levels in lymphocyte-deficient mice (Brachman et al., 2015). Interpretation of these results is that stress confers an “immunological memory” through alterations within the adaptive immune cell compartment thus inoculating against future stress exposure. Future therapeutics may be designed towards adapting T cell-mediated responses to promote resilience (Toben and Baune, 2015).

Interestingly, it is possible that some of the therapeutic effects of standard antidepressant are through their actions on T cells. For example, administration of fluoxetine, an antidepressant that acts by blocking serotonin reuptake, normalized stress-induced reductions in T cell reactivity and proliferation (Frick et al., 2009). Moreover, chronic treatment with desipramine and fluoxetine were able to attenuate a T-cell dependent contact hypersensitivity response in mice, and this effect required the presence of CD8 T cells and natural killer cells (Curzytek et al., 2015). In humans, one study recently showed that clinically significant therapeutic responses to antidepressants were associated with increased circulating levels of regulatory T cells (Himmerich et al., 2010). Together, studies in preclinical stress models and human depression support the idea that T cells hold the capacity to inoculate an individual against stress and promote behavioral resilience by lowering systemic inflammation. Lifestyle changes or therapeutics designed to boost the body’s natural immune defenses represent an important new direction in resilience research.

#### 4.4.4. Pro-resilience effects of natural compounds

Throughout history, natural compounds have been the source for discovery of active ingredients isolated and utilized to derive powerful pharmaceuticals drugs. As such, this has spurred active investigations into natural compounds that promote resilience in an attempt to discover new antidepressant therapeutics. One of the most studied categories of natural compounds are polyphenols found in large part in grapes and grape-derived products (Xu et al., 2011). Polyphenols exhibit strong antioxidant, anti-inflammatory, antimicrobial, and anti-tumorigenic activities (Auger et al., 2016; Gollucke et al., 2013; Petersen and Smith, 2016). Recent evidence indicates that grape-derived natural compounds may promote stress resilience in both males and females by inhibiting key inflammatory processes in the periphery. For example, a cocktail of malvidin-3′-*O*-glucoside (Mal-gluc) and dihydrocaffeic acid (DHCA), two bioactive polyphenol metabolites identified from grape-derived products, reduced the release of leukocyte-derived IL-6 though a DNA-methylation dependent epigenetic mechanism (Wang et al., 2018). Notably, DHCA was also found to have strong inhibitory properties on other key inflammatory cytokines such as IL1β and IL-12. One of the best-studied polyphenols, resveratrol, has both antidepressants effects in the social defeat stress model (Finnell et al., 2017; Renthal et al., 2009) and pro-cognitive effects in models of stress-related cognitive impairments (Solanki et al., 2017). In part, the pro-resilient effects of resveratrol are thought to be mediated by up-regulation of a sirtuin 1 (SIRT1)-dependent pathway (Kim et al., 2016) that can epigenetically reprogram oxidative stress and pro-inflammatory gene expression profiles (Strycharz et al., 2017). A greater understanding of how these natural compounds act to promote resilience could help in developing stress coping strategies focused on dietary nutrients.

## 5. Influence of the microbiota-gut-brain axis on resilience

The influence of immune factors on resilience can no longer be examined independently from the way adversity impacts the gastro-intestinal microbiota and conversely, how this microbiota influences immune and behavioral responses involved in the resilience process. In this section, we will examine the mechanisms by which the commensal gut microbiota regulates neuroinflammation and in this way influences the risk for depression, seen as a failure of the resilience process in face of adversity.

### 5.1. Microbiota-gut-brain axis

**Fig. 4:**
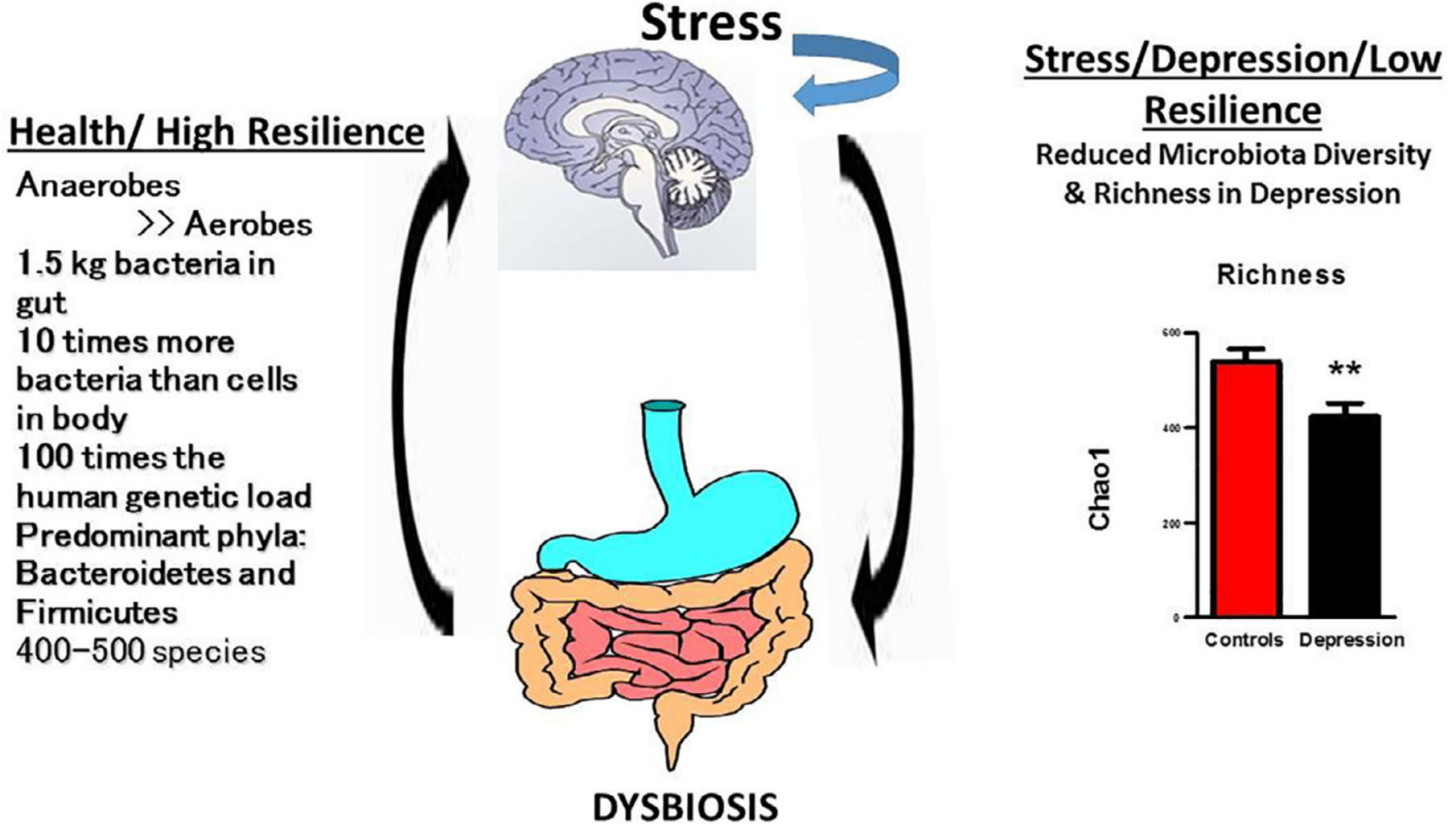
Role of the gut microbiota in gut-to-brain signaling in health with high resilience and in depression with an exaggerated stress response. The communication between gut and brain is bidirectional. Stress can lead to gut dysbiosis and in turn dysbiosis can result in central changes leading to decreased resilience with vulnerability to depression. As can be seen, Chao 1 species richness is decreased in depression.

Over the past decade the role of the microbiota-gut-brain axis in modulating resilience and response to stress has been increasingly studied (Fig. 4). In fact, the approach has been described as a new paradigm in neuroscience (Mayer et al., 2014). For decades, the brain-gut axis was studied by physiologists and the concept of a ‘second brain’, because of the sheer number of neurons in the gut wall, gained widespread acceptance. However, it is only recently that the role of bacteria within the brain-gut axis has been investigated (Dinan and Cryan, 2017). Previously, commensal bacteria were not considered to have any major role to play in brain function. We now know from germ free studies that gut microbes play a pivotal role in establishing the blood brain barrier (BBB), in neuronal myelination, and in the functioning of key neurotransmitter systems such as serotonin (Luczynski et al., 2016). Intriguingly, from an evolutionary perspective, it has been demonstrated that microbes are capable of synthesizing all of the major neurotransmitters found in the human brain (Patterson et al., 2014). For example, lactobacilli synthesize GABA and other microbes synthesize serotonin, noradrenaline and dopamine. However, apart from neurotransmitter production the gut microbiota acts as a fermenter which produces a vast array of compounds of importance to a variety of organs including the brain. The aim of this section on the microbiota-gut-brain axis is not to examine all the human and animal studies that have been carried out in the field, but to provide a few examples of these studies to illustrate their implication for the relationship between immunity and resilience

### 5.2. Microbiota-gut-brain communication

There are a number of parallel routes through which gut microbes communicate with the brain (Dinan and Cryan, 2015) and in turn the brain communicates with the gut. The vagus nerve is a key channel for information transfer. Bravo et al showed that in rodents a lactobacillus strain of bacteria (JB1) had an impact on behavior acting through GABA receptors in various brain regions (Bravo et al., 2011). However, when animals were vagotomised, no such effects were observed. Short chain fatty acids such as butyrate and proprionate are the products of bacterial metabolic activity. These can act through G-protein coupled receptors (GPCRs) and also as epigenetic modulators, inhibiting histone deacetylase (HDAC) (Dinan and Cryan, 2017). It has been speculated that they reach the brain via the blood stream but this has yet to be clearly demonstrated using physiologically relevant doses. Tryptophan from the diet has long been known to regulate central serotoninergic neurotransmission. However, it has now been shown that bifidobacteria within the intestine can synthesize tryptophan and that administration of bifidobacteria is associated with increased plasma levels of tryptophan (Desbonnet et al., 2008). Tryptophan produced by the gut microbiota or derived from food can be metabolized by IDO1 that is present in the gut mucosa, leading to increased blood levels of kynurenine. Kynurenine crosses the gut-blood barrier and can get into the brain via the blood stream. Some microbial strains such as *Lactobacillus reuteri* can down-regulate IDO1 in the gut mucosa and in this way modulate circulating kynurenine levels (Marin et al., 2017). Pro-inflammatory cytokines produced by immune cells in the gut mucosa in response to certain strains of gut bacteria can activate the hypothalamic-pituitary-adrenal axis, most notably IL-1 and IL-6, and in certain circumstances can signal to the brain via the multiple pathways of communication already described in a previous section. Of note, increased activation of the hypothalamic-pituitary-adrenal axis also increases circulating levels of kynurenine via activation of the hepatic tryptophan 2,3 dioxygenase (TDO2).

### 5.3. Components of gut microbiota

There are large inter-individual differences in the microbiota of healthy adults, even between monozygotic twins, but nonetheless a shared core gut microbiome exists, exerting a common functionality within the host. The adult microbiota is dominated by members of the *Bacteroidetes* and *Firmicutes* phyla (Turnbaugh et al., 2006). In adulthood, diet is the main determinant of the gut microbiota composition, although many drugs and especially antibiotics can dramatically alter composition. A diverse diet rich in fiber, especially prebiotic fibers such as inulin, is associated with microbial diversity. In marked contrast, a diet of highly processed fast food is associated with limited diversity. Drugs, especially antibiotics, negatively impact microbial variety and can induce a dysbiosis. This impact may be greater at the extremes of life in both neonates and the elderly. Regular aerobic exercise which is increasingly recognized as promoting stress resilience also promotes greater diversity (Barton et al., 2017).

### 5.4. Gut microbiota and depression

There is accumulating evidence that some psychiatric disorders are associated with a gut dysbiosis. Using a maternal separation model of depression in rats (O’Mahony et al., 2009) it was shown that adult animals subjected to this paradigm as pups had a less diverse micro-biota than animals who were raised without stress. The less diverse microbiota was associated with an exaggerated response of the hypothalamic-pituitary-adrenal axis, increased pro-inflammatory cytokines and overall decreased exploratory behavior, characterized as anxiety. In another model of stress, mice submitted to chronic mild stress for 5 weeks developed depression-like behavior that was associated with decreased levels of *Lactobacillus* (Marin et al., 2017). The development of depression-like behavior was dependent on stress-induced increases in circulating levels of kynurenine and could be treated by administration of *Lactobacillus reuteri* which, as mentioned earlier, down regulated IDO1 activity.

In accordance with preclinical findings, clinical studies also show evidence of gut dysbiosis in depressed patients. For instance, Jiang et al. (2015) analysed faecal samples from 46 patients with major depression and 30 healthy controls. Patients acutely depressed had higher levels of *Bacteroidetes, Proteobacteria* and *Actinobacteria,* whereas levels of *Firmicutes* were significantly reduced. A negative correlation was observed between *Faecalibacterium* and the severity of depressive symptoms. A study conducted in APC Microbiome Ireland (Kelly et al., 2016) found that depression is associated with decreased gut microbiota richness and diversity. When a fecal microbiota transplantation from depressed patients into microbiota-depleted rats was carried out, it induced behavioral and physiological features characteristic of depression in the recipient animals, including anhedonia and anxiety-like behaviours, as well as increased metabolism of tryptophan into kynurenine, probably as a consequence of IDO1 activation, together with increases in the acute phase protein CRP. This suggests that the gut microbiota may play a causal role in the development of mood disorders or at least profoundly impact resilience.

### 5.5. Development of psychobiotics

The concept of a probiotic was introduced in the literature by the Nobel Laureate Metchnikoff, who observed the fact that individuals living in a region of Bulgaria who drank large amounts of fermented milk had an extended life span. Probiotics are generally defined as live bacteria which have a positive health benefit, while psychobiotics are defined as bacteria which when ingested in adequate amounts have a positive mental health benefit and promote psychological resilience (Dinan et al., 2013). More recent publications, under the heading of psychobiotics, have included prebiotic fibers, which promote the growth of ‘good’ bacteria (Sarkar et al., 2016).

While much of the work on psychobiotics is at a pre-clinical level, there are human interventions supporting the view that psychobiotics increase resilience. Most putative probiotics have no impact on behavior and identifying strains with psychobiotic potential is challenging (Bambury et al., 2017). In preclinical studies, the *Bifidobacterium longum* 1714 strain was found to improve stress-related behaviors and cognitive performance. A follow on human intervention study confirmed these findings (Allen et al., 2016). In a within-participants design, healthy volunteers completed cognitive assessments, resting electroencephalography and were exposed to a socially evaluated cold pressor test at baseline, post-placebo and post-psychobiotic. Increases in salivary cortisol output and subjective anxiety in response to the socially evaluated cold pressor test were attenuated by the psychobiotic. Furthermore, daily stress reported by the subjects was reduced by psychobiotic consumption. Subtle improvements in hippocampus-dependent visuospatial memory performance were detected as well as enhanced frontal midline electroencephalographic mobility. These clear benefits are in line with the predicted impact from preclinical screening platforms. The data indicate that consumption of *B. longum* 1714 is associated with decreased stress sensitivity and improved memory.

In another healthy volunteer study, Messaoudi and colleagues conducted a placebo-controlled parallel group design study in healthy volunteers. Subjects were randomly assigned to receive either a combination of *Lactobacillus helveticus* R0052 and *Bifidobacterium longum* or matching placebo for 30 days (Messaoudi et al., 2011). On a variety of stress measures the psychobiotic treatment had a positive impact. Furthermore, urinary free cortisol output was reduced by the psychobiotics, again supporting the view that psychobiotics can impact the hypothalamic-pituitary-adrenal axis and thereby impact resilience.

The effect of *Lactobacillus rhamnosus* HN001 given in pregnancy and postpartum on symptoms of maternal depression and anxiety in the postpartum period was assessed (Slykerman et al., 2017). Two hundred and twelve women were randomized to HN001 and 211 to placebo. Women who received HN001 had significantly lower depression and anxiety scores in the postpartum period. The results strongly support the view that the psychobiotic is protective against the emergence of postpartum symptoms. The postpartum period is recognized as a time when resilience is at its lowest levels and the capacity to impact this with psychobiotics is of major significance.

### 5.6. Diet, microbes and depression

Diet is a major bridge between the gut microbiota and resilience. Poor quality diet reduces resilience and is a risk factor for major depression. Diets rich in fruit, vegetables, grains and fish seem protective against depression while a diet of highly proceeded foods predispose to depression. A recent study from Australia used a randomized controlled trial (RCT) design to investigate the efficacy of a dietary program for the treatment of major depression (Jacka et al., 2017). A structured dietary support, focusing on improving diet quality using a modified Mediterranean diet was compared to a social support control condition. Sixty-seven patients were recruited fulfilling criteria for major depression and scoring 75 or less, out of a possible score of 104, on a Dietary Screening Tool, a score which indicated a poor baseline diet. If patients were on antidepressant medication or undergoing psychotherapy, they were required to be on the same treatment for at least 2 weeks prior to study entry. The dietary intervention group showed a significantly greater improvement in depression scores between baseline and 12 weeks than the social support control group. Overall, the results of this trial suggest that improving diet may be a useful strategy for treating depression or at least as an adjunctive to conventional therapies. It has long been recognized that those on a Mediterranean diet show increased resilience and are less susceptible to depression, but the recent study indicates that such a diet can have a therapeutic benefit when an individual becomes depressed (Carlos et al., 2018; Opie et al., 2017). However, we do not know which components of the Mediterranean diet play the most significant role from a mental health perspective. It might be the high levels of prebiotics, the high levels of polyphenols or the high intake of polyunsaturated fatty acids. The latter are largely acquired from fish and while clearly being of structural importance in the brain are also known to act as prebiotics, increasing the levels of good bacteria.

### 5.7. Summary

There is evidence that decreased diversity of the microbiota negatively impacts resilience and increases the risk of depression/anxiety. Undoubtedly, poor diet and antibiotic exposure can lead to this state of dysbiosis with resultant vulnerability. Studies in healthy volunteers indicate that certain psychobiotics can improve resilience and may help prevent depression, while a modified Mediterranean diet may exert antidepressant effects probably also by acting through the microbiota. The impact of diet has traditionally been ignored by both psychiatrists and clinical psychologists but nutritional psychiatry is now emerging as an important field.

## 6. Conclusions

Based on the human and animal studies that are described in this paper, it should be apparent that the psychological qualities that are part of the resilience process have the ability to attenuate the adverse effects of stress on immunity, particularly inflammatory responses, and vice versa that immune factors can influence the way individuals adapt to stressful situations and recover from them. However, this very general statement masks a number of important gaps in the literature that, once identified, could help defining a more elaborate research agenda on resilience and immunity than what has been done so far. In particular, it is clear that human research on resilience and immunity is still lagging behind. Psychological factors that are part of the resilience process have been examined independently from each other, and the use of different outcomes in different studies does not facilitate integration of the data.

There is also a lack of research on facets of the resilience process such as the provision of information about stressors, coping strategies, coping resources, and opportunities to express stress-associated emotions. These processes should be at the core of resilience and account for substantial variance in the role of positive factors in protecting against stressor-induced alterations in immunity. In addition, studies on psychological factors should be complemented by investigation of the adaptive purposes of the resilience factors and of the specific attitudes and behaviors taken to fulfill these purposes. Social support is a typical example, with the effects of inflammation on interpersonal behaviors being complex but still consistent in purpose. As detailed in Section 4.3, studies of social behavior in inflamed individuals reveal that inflammatory processes help individuals to navigate the social environment during times of inflammation-induced sickness (Eisenberger et al., 2017). More specifically, inflammation increases sensitivity to negative or threatening social experiences while at the same time enhancing sensitivity to positive social experiences. Knowing that inflammation is enhanced in individuals who have experienced childhood adversity, it is easy to understand the adaptive nature of this mutual relationship between inflammation and sociability in an individual’s life trajectory (Nusslock and Miller, 2016).

The effects of inflammation are not specific to social stimuli. There is evidence that these effects are just another facet of the ability of inflammation to make individuals more finicky by enhancing the perceived contrast between stimuli of different hedonic valence (Aubert and Dantzer, 2005; Vichaya et al., 2014). In other words, inflammation has the potential to modulate appraisal of adversity. However, it is important to mention that most of the data that are available on the effects of inflammation on behavior, mood and emotional regulation have been obtained in very specific situations in which the inflammatory cytokine cascade is activated by stressors or by injection of pathogen-associated molecular patterns, e.g., endotoxin. The way this can generalize to other situations is still unknown.

An often-neglected issue in most studies on the relationship between stress and depression on the one hand and immunity on the other hand is the potential role of the unhealthy lifestyles and behaviors that are often associated with such conditions as mechanisms linking adversity to immune function. Conversely, most studies on positive factors do not account for the likely influence of health practices including diet and hygiene. Although these lifestyle factors may play a causal role in both the level of a resilience factor and the immune response, they may also act as mediating pathways through which a resilience factor influences stress effects on immunity. In view of the role played by the gut microbiota and probably by other microbiota including the oral and skin microbiota, these issues are not trivial.

Last but not the least, it should be clear from the examined literature that the psychological and biological factors that promote adaptation to adverse situations have been given much more emphasis than the factors that promote the recovery process. As mentioned several times in this review, resilience is not just the capacity to adapt but also the ability to bounce back and gain from the experience of adversity, something for which resolution of the detrimental effects of the situation is a prerequisite. As immune processes and adjustment behaviors powerfully regulate each other, the possibility of an interplay between the innate immune system and the adaptive immune system in the recovery process clearly requires further consideration. This aspect brings back onto the stage the time factor that is unfortunately often neglected in the field of resilience and immunity as cross-sectional studies largely predominate over longitudinal studies.

## Figures and Tables

**Table 1:** Key aspects of the resilience process in human and animal studies. Items highlighted in gray are those that are detailed in the present review.

Components of the resilience process	Human studies	Animal studies
Adverse conditions	Always present	Always present (fixed by the experimenter)
Individual attributes	Early life experience	Not examined
	Personal control	
	Positive affect	
	Optimism	
	Hardiness	
Environmental characteristics	Social networks	Not examined
	Current socioeconomic conditions	
Outcomes	Absence of a negative outcome (buffering of the effect of adversity on psychopathology, physical illness, immune function)	Individual differences in the ability to behaviorally cope with the adverse conditions (define resilience versus sensitivity)
	Cost of resilience, allostatic load	Biological correlates of individual differences, recovery process
	Positive outcome (e.g, mastering, social competence)	Not examined
Risk factors compromising resilience	Physical/sexual abuse	Not examined
	Low socioeconomic status during early life	Early life stress including infection
Intervention	Psychological	Not examined
	Immunological	Immunological intervention including vaccination
	Nutritional	Nutritional
